# Interrelationships Between Pituitary Hormones as Assessed From 24-hour Serum Concentrations in Healthy Older Subjects

**DOI:** 10.1210/clinem/dgz253

**Published:** 2019-12-19

**Authors:** Evie van der Spoel, Ferdinand Roelfsema, Abimbola A Akintola, Steffy W Jansen, P Eline Slagboom, Rudi G J Westendorp, Gerard J Blauw, Hanno Pijl, Diana van Heemst

**Affiliations:** 1 Section Gerontology and Geriatrics, Department of Internal Medicine, Leiden University Medical Center, RC Leiden, The Netherlands; 2 Section Endocrinology, Department of Internal Medicine, Leiden University Medical Center, RC Leiden, The Netherlands; 3 Section Molecular Epidemiology, Department of Biomedical Data Sciences, Leiden University Medical Center, RC Leiden The Netherlands; 4 Department of Public Health, Center of Healthy Aging, University of Copenhagen, Copenhagen, Denmark

**Keywords:** human, pituitary hormones, time series data, cross-correlation, cross-approximate entropy, familial longevity

## Abstract

**Context:**

Hormones of the hypothalamic-pituitary-target gland axes are mostly investigated separately, whereas the interplay between hormones might be as important as each separate hormonal axis.

**Objective:**

Our aim is to determine the interrelationships between GH, TSH, ACTH, and cortisol in healthy older individuals.

**Design:**

We made use of 24-hour hormone serum concentrations assessed with intervals of 10 minutes from 38 healthy older individuals with a mean age (SD) of 65.1 (5.1) years from the Leiden Longevity Study. Cross-correlation analyses were performed to assess the relative strength between 2 24-hour hormone serum concentration series for all possible time shifts. Cross-approximate entropy was used to assess pattern synchronicity between 2 24-hour hormone serum concentration series.

**Results:**

Within an interlinked hormonal axis, ACTH and cortisol were positively correlated with a mean (95% confidence interval) correlation coefficient of 0.78 (0.74–0.81) with cortisol following ACTH concentrations with a delay of 10 minutes. Between different hormonal axes, we observed a negative correlation coefficient between cortisol and TSH of -0.30 (-0.36 to -0.25) with TSH following cortisol concentrations with a delay of 170 minutes. Furthermore, a positive mean (95% confidence interval) correlation coefficient of 0.29 (0.22–0.37) was found between TSH and GH concentrations without any delay. Moreover, cross-approximate entropy analyses showed that GH and cortisol exhibit synchronous serum concentration patterns.

**Conclusions:**

This study demonstrates that interrelations between hormones from interlinked as well as different hypothalamic-pituitary-target gland axes are observed in healthy older individuals. More research is needed to determine the biological meaning and clinical consequences of these observations.

Hormones of the hypothalamic-pituitary-target gland axes are regulated by central and peripheral feedforward and feedback signals. The interplay among these regulators in time dictates the hormone secretion pattern, which will be adapted depending on the changing needs of the organism, such as during the circadian rhythm, sleep, activity, food intake, stress, and inflammation. Although hormones of the hypothalamic-pituitary-target gland axes are each regulated by different factors and respond to different signals, the common goal of all these hormonal axes is to maintain homeostasis in the human body. Furthermore, anterior pituitary cells share the same embryonic origin and there is evidence for crosstalk between pituitary cells (1–4). These shared features, however, have rarely been addressed in human studies, whereas the interplay between hormones might be as important, or more important, as each separate hormonal axis. For example, with aging or after menopause, levels of several hormones change concomitantly. While this might reflect separate mechanisms, these hormonal changes could also be synchronized with each other and their concerted impact might be larger than the sum of their individual impact on the aging phenotype. Also, in other systems and organs of the body, interplay, interaction, and networks are highly important for maintenance of homeostasis and proper functioning of the human body.

Little is known about the interrelationships of hormones from different hypothalamic-pituitary-target gland axes, especially over time because patients, or healthy individuals, are rarely sampled multiple times during the day. Some studies did collect hormonal time series data and investigated associations between pituitary hormones and/or hormones from an endocrine target gland. For example, in patients with Cushing syndrome, who display excessive production of cortisol, pulsatile TSH secretion is suppressed and irregular (5). TSH secretion is also decreased in patients with acromegaly who display excessive production of GH (6). However, these studies were performed in patients, so the observed relationships could be influenced by other aspects of their illness and not only by the altered hormone secretion. Few studies have been performed in healthy individuals. For example, Vis *et al.* assessed hormonal relationships in 18 obese women and found among others relationships between ACTH and cortisol, TSH and GH, TSH and cortisol, and between TSH and ACTH (7). Furthermore, glucocorticosteroid administration directly suppressed pulsatile TSH secretion in healthy men (8) and a positive cross-correlation between GH and cortisol was found in older men and women (9).

In the present study, we aimed to determine the interrelationships between GH, TSH, ACTH, and cortisol in healthy older men and women. To this end, we analyzed 24-hour hormone concentration series assessed at intervals of 10 minutes from 38 healthy older participants from the Leiden Longevity Study (10). Moreover, we examined whether interrelationships between hormones differ between men and women or between offspring of long-lived families and their partners. Furthermore, differences between interrelationships during the lights-on and lights-off periods were determined. We performed cross-correlation analyses to assess the relative strength between 2 24-hour hormone concentration series for all possible time shifts and cross-approximate entropy (ApEn) to assess pattern synchronicity between the different 24-hour hormone concentration series.

## Methods

### Study participants

In the Switchbox Leiden Study, we collected 24-hour blood samples from 38 healthy older (range, 52–76 years) individuals comprising 20 men and 18 women between June 2012 and July 2013 (11). Participants were recruited from the Leiden Longevity Study, which is a family-based study consisting of 421 families with at least 2 long-lived siblings (men ≥ 89 years and women ≥ 91 years) together with their offspring and the offspring’s partners without any selection on health or demographics (12). The Switchbox Leiden Study comprised 20 offspring of long-lived families, including 10 men and 10 women, and 18 partners of the offspring as a control group, including 10 men and 8 women. Participants had a stable body mass index (BMI) between 20 and 34 kg/m^2^ and although not formally asked, based on the age range, the majority of women was most likely postmenopausal. Exclusion criteria were having a fasting plasma glucose above 7 mmol/L, having chronic renal, hepatic, or endocrine disease, or using medication known to influence lipolysis, thyroid function, glucose metabolism, GH or IGF-1 secretion, and/or any other hormonal axis. Hence, none of the participants were using estrogen-containing compounds. Participants were excluded if they had a recent trans-meridian flight or when they recently performed shift work. To be able to safely perform the 24-hour blood sampling, other exclusion criteria were difficulties to insert and maintain an intravenous catheter, anemia (hemoglobin < 7.1 mmol/L), and blood donation within the last 2 months. None of the participants indicated that they were using any biotin or vitamin B8 supplements, which otherwise could have interfered with the ACTH assay (13). The Switchbox Leiden Study protocol was approved by the Medical Ethical Committee of the Leiden University Medical Centre and performed according to the Helsinki declaration. All participants gave written informed consent for participation.

### Study protocol

Participants were admitted to the Research Centre at 0800 hours, where a catheter was placed in a vein of the forearm of the nondominant hand. Blood sampling started around 0900 hours and every 10 minutes, 2 mL of blood was collected in a serum tube and 1.2 mL in an EDTA tube (10). The participants received standardized feeding consisting of 600 kcal Nutridrink (Nutricia Advanced Medical Nutrition, Zoetermeer, The Netherlands) at 3 fixed times during the day (between 0900 and 1000 hours, 1200 and 1300 hours, and 1800 and 1900 hours). Lights were switched off between 2300 and 0800 hours to allow the participants to sleep and except for lavatory use, no physical activity was allowed during the study period. All participants were sampled in the same research room. Anthropometric measurements, comprising weight, height, waist circumference, fat mass, and lean body mass were performed in the Research Centre using a scale, measuring tape, and Bioelectrical Impedance Analysis at a fixed frequency of 50 kHz (Bodystat 1500 Ltd., Isle of Man, UK). BMI was calculated as weight (in kilograms) divided by the square of height (in meters). Data on habitual bedtime and getting up time during the past month were obtained using the Pittsburgh Sleep Quality Index questionnaire (14).

### Biochemical assays

All laboratory measurements were performed with fully automated equipment and diagnostics from Roche Diagnostics (Almere, The Netherlands) and Siemens Healthcare diagnostics (The Hague, The Netherlands) at the Department of Clinical Chemistry and Laboratory Medicine of the Leiden University Medical Center in The Netherlands. Full details on the procedures of the hormone assays have been described previously (11,15,16). Levels of GH, TSH, cortisol, and ACTH were all measured in blood samples collected every 10 minutes from all 38 participants. For each participant, all samples from 1 time series were measured with the same lot number in the same batch. Human GH with a molecular mass of 22 000 Da (Siemens, Cat# L2KGRH2, Research Resource Identifier [RRID]: AB_2811291) was measured in serum samples using an IMMULITE 2000 Xpi Immunoassay system (Siemens Healthcare Diagnostics) (17). TSH (Roche, Cat# 11731459, RRID:AB_2756377) and cortisol (Roche, Cat# 11875116, RRID:AB_2811288) were measured in serum samples by ECLIA (ElectroChemoLuminescence ImmunoAssay) using Cobas reagents and a Roche Modular E170 Immunoanalyzer (18,19). ACTH (Siemens, Cat# L2KAC2, RRID:AB_2783635) was measured in EDTA samples using an IMMULITE 2000 Xpi Immunoassay system (Siemens Healthcare Diagnostics) (20). The data were checked for obvious outliers by visual inspection of a graphical display of individual hormone profiles from all 38 participants (21). This was performed by 4 reviewers with expert knowledge in endocrinology. After reviewing the data individually, a consensus meeting was held to reach agreement on data points which only 1 or 2 reviewers had marked as an outlier. For 28 of 38 participants, 1.1 (SD = 1.8) data points per hormonal concentration series were on average detected as outliers and excluded from the dataset. Glucose and insulin were measured in a fasting serum sample withdrawn around 0830 hours at the second day of the 24-hour blood sampling. Glucose was measured using Roche Hitachi Modular P800 and insulin was measured using IMMULITE 2000 Xpi Immunoassay.

### Cross-correlation

Cross-correlation assesses the relative strength between 2 24-hour hormone concentration series for all possible time shifts, by calculating linear Pearson’s correlation coefficients as explained in more detail elsewhere (22,23). For example, hormone concentrations in time series A are compared pairwise with those of series B measured simultaneously (0 lag) or measured earlier or later (with a time lag). The unit of 1 lag time is the interval between 2 sampling points, so a lag time of 1 means that there is a delay of 10 minutes between 2 time series. Cross-correlation analyses were performed using the ccf function in the software program R, version 3.4.3 (The R Foundation for Statistical Computing, Vienna, Austria). The range of tested lag times depends on the number of data points in one time series; the range is lag -18 to 18 (360 minutes in total) for 144 data points. A correlation is considered significant when the absolute value is greater than 2/(√n–│k│), where n is the number of data points in 1 time series and k is the maximal possible lag (24). For a time series of 144 data points and a maximal lag of 18, the significance level is 0.18. Cross-correlation analyses were also performed after stratifying the 24-hour data for the lights-on period, which are the data from time point 0900 hours up to and including 2250 hours, and lights-off period (2300 to 0800 hours). For these subanalyses, the lag range and the significance level changed accordingly to a lag range of -16 to 16 (320 minutes) and significance level of 0.24 for the lights-on period, and a lag range of -14 to 14 (280 minutes) and significance level of 0.31 for the lights-off period.

### Cross-ApEn

Bivariate cross-approximate entropy (cross-ApEn) quantifies joint pattern synchrony between 2 simultaneously measured time series, with lower cross-ApEn values signifying greater synchrony (25,26). Synchrony refers to pattern similarity, so to what extent subpatterns co-occur both in time series A and in time series B. Cross-ApEn was calculated with a window length of *m =* 1 and a margin of *r =* 0.2 (20% of the SD of the individual subject’s hormone time series) with standardized data using the Matlab software program (Mathworks, Inc., Natick, MA). Subsequently, jackknifing was performed, which is a rigorous and objective leave-1-out cross-validation test that gives less bias in smaller samples than regular cross-ApEn, and it is more applicable for hormone data. It is important to note that a cross-ApEn of hormones A-B is different from a cross-ApEn of hormones B-A because A is leading in the first case and following in the second. Cross-ApEn analyses were also performed after stratifying the 24-hour data for lights-on period, which are the data from time point 0900 hour up to and including 2250 hours, and lights-off period (2300 to 0800 hours). Because cross-ApEn analyses cannot deal with missing data, missing data points were linearly interpolated.

### Statistical analysis

Characteristics of the study participants were calculated using descriptive statistics. Normally distributed variables were presented as mean with SD and differences between men and women and between offspring and partners were assessed by independent-samples *t*-tests. Not normally distributed variables were presented as median with interquartile ranges, using the nonparametric independent-samples Mann-Whitney *U* test to assess differences between subgroups. All statistical analyses were performed using SPSS for Windows, version 23 (SPSS, Chicago, IL). Tukey box plots were made using GraphPad Prism version 7 (GraphPad, San Diego, CA).

## Results

### Characteristics of study participants

Characteristics of study participants are presented in Table 1 for all participants and stratified for sex and offspring-partner status. The number of men was equal in offspring and partner groups. Men and women were also similar in their offspring-partner distribution. Participants had a mean (SD) age of 65.1 (5.1) years, which was similar for men and women and for offspring and partners. The observed median (interquartile range) BMI of 24.8 (22.2–28.0) kg/m^2^ is normal for this age category and was similar for all subgroups. Fasting glucose and insulin levels were for all participants within the reference range of our laboratory, with similar levels between groups. As expected, men and women differed in measures of body composition, with men being taller, having less fat mass, more lean body mass, and larger waist circumference than women. Groups of offspring and partners were similar in body composition. Participants were normal nocturnal sleepers in the month prior to the study day with a median (interquartile range) habitual bedtime of 2330 (2300–2400) hours and getting up time of 0800 (0730–0815) hours, which is similar to the time schedule of the study protocol during the 24-hour blood sampling. Habitual bedtimes and getting up times were similar for men and women and for offspring and partners.

**Table 1.** Characteristics of Study Participants, for All Subjects and Stratified for Sex and Family History

		Stratified for Sex			Stratified for Family History		
	All subjects (n = 38 )	Men (n = 20 )	Women (n = 18 )	*P* Value	Offspring (n = 20 )	Partners (n = 18)	*P* Value
Male, N (%)	20 (52.6)	NA	NA	NA	10 (50)	10 (55.6)	0.76
Offspring of long-lived family, N (%)	20 (52.6)	10 (50)	10 (55.6)	0.76	NA	NA	NA
Age (y)^a^	65.1 (5.1)	65.6 (5.3)	64.6 (5.0)	0.56	65.6 (5.4)	64.6 (4.9)	0.52
BMI (kg/m^2^)^b^	24.8 (22.2–28.0)	25.2 (23.3–27.4)	23.1 (21.6–29.9)	0.21	24.8 (22.3–29.3)	25.1 (22.1–27.7)	0.96
Height (cm)^b^	175 (165–181)	178 (175–182)	165 (162–168)	<0.001	175 (164–180)	175 (167–182)	0.58
Fat mass (kg)^b^	20.5 (18.5–27.0)	19.1 (18.0–24.1)	23.5 (19.7–34.7)	0.02	21.9 (18.7–27.5)	20.4 (18.4–29.1)	0.78
Lean body mass (kg)^b^	53.3 (41.5–62.2)	60.5 (57.6–66.0)	41.5 (37.4–44.8)	<0.001	52.4 (41.8–62.8)	54.3 (40.4–63.0)	0.73
Waist circumference (cm)^b^	94 (82–100)	97 (92–106)	82 (80–95)	0.001	92 (82–101)	94 (83–98)	0.96
Fasting glucose (mmol/L)^a^	4.9 (0.6)	4.9 (0.7)	4.9 (0.5)	0.98	4.9 (0.7)	4.8 (0.4)	0.51
Fasting insulin (mU/L)	5.7 (3.7–7.9)	6.2 (3.4–10.1)	5.1 (3.9–6.3)	0.44	4.5 (3.5–8.0)	5.9 (3.8–7.8)	0.78
Habitual bedtime (h)	23:30 (23:00–00:00)	23:30 (23:00–23:45)	23:30 (23:00–23:45)	0.68	23:30 (23:00–00:00)	23:30 (23:00–23:30)	0.92
Habitual getting up time (h)	08:00 (07:30–08:15)	07:45 (07:00–08:15)	08:00 (07:30–08:15)	0.23	08:00 (07:30–08:30)	07:45 (07:00–08:15)	0.35

Unless indicated otherwise, data are presented as median with interquartile ranges. ^a^Data are presented as mean with SD. ^b^Data were not available for 1 male partner. NA, not applicable.

### Hormone concentration profiles over 24 hours

**Figure 1.**
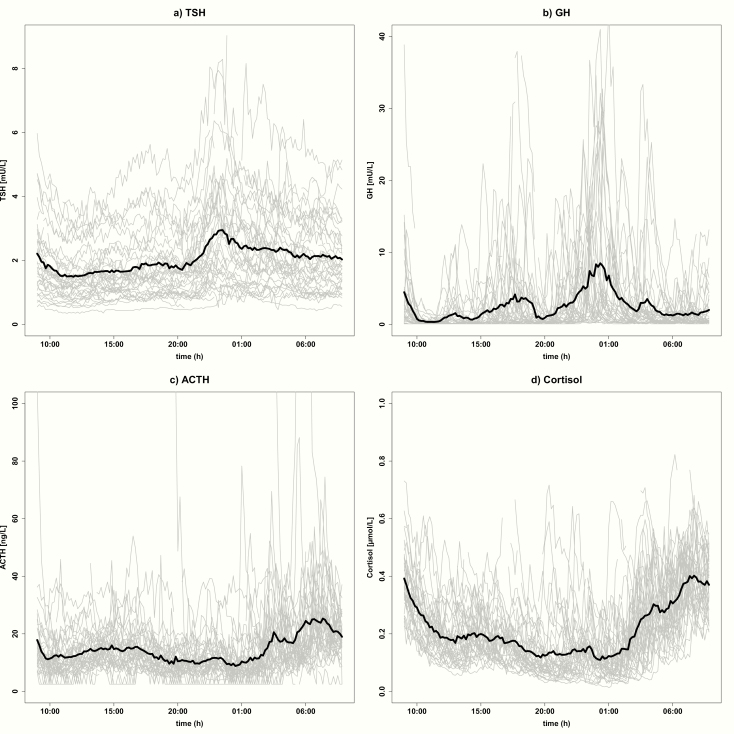
Hormone concentration profiles over 24 hours for (A) TSH, (B) GH, (C) ACTH, and (D) cortisol. For each hormone, the hormone concentration profiles over 24 hours are depicted for each of the 38 individual participants as gray lines. For each of the hormones, the mean hormone concentration profile of the 38 participants over 24 hours is depicted as a black line. Serum hormone concentration measurements were performed every 10 minutes for 24 hours, starting around 09:00 hours.

The hormone concentration profiles over 24 hours are plotted for each of the 38 individual participants and illustrated in Fig. 1 for TSH (A), GH (B), ACTH (C), and cortisol (D). Furthermore, for each of the hormones, the mean hormone concentration profile of the 38 participants over 24 hours is depicted. For GH, we observed a wide variation between individual 24-hour GH profiles. Separate plots of individual GH concentration profiles can be found elsewhere (16).

### Cross-correlations of GH, TSH, ACTH, and cortisol

**Figure 2.**
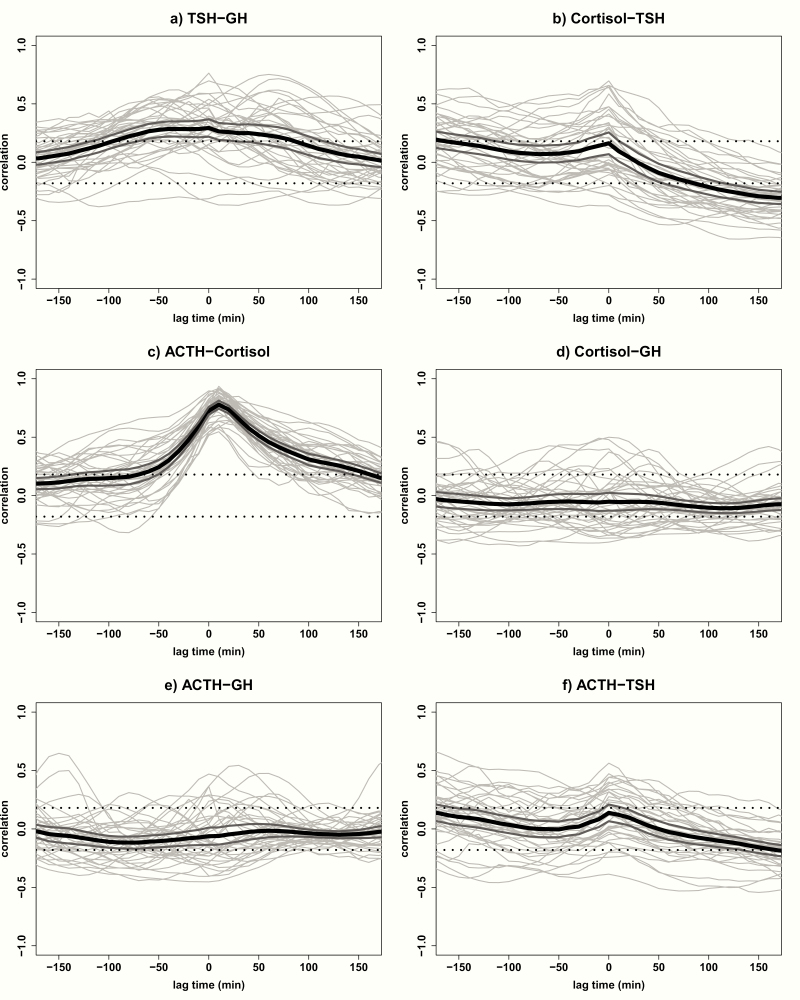
Cross-correlations of GH, TSH, ACTH, and cortisol. Cross-correlations of (A) TSH and GH, (B) cortisol and TSH, (C) ACTH and cortisol, (D) cortisol and GH, (E) ACTH and GH, and (F) ACTH and TSH in all 38 participants. Cross-correlation assesses the relative strength between two hormone time series for all possible time shifts. The graph displays the correlation (y-axis) at a lag time in minutes (x-axis) with each gray line corresponding with 1 participant. The black line indicates the mean correlation for all participants and the 2 dark gray lines indicate the 95% confidence interval. The significance level is indicated by 2 straight dotted lines at correlations -0.18 and +0.18. Negative lag times represent a correlation in which hormone 2 is followed by hormone 1; positive lag times represent a correlation in which hormone 1 is followed by hormone 2.

Figure 2 presents the cross-correlations between TSH and GH (A), cortisol and TSH (B), ACTH and cortisol (C), cortisol and GH (D), ACTH and GH (E), and ACTH and TSH (F) in all 38 participants. For TSH and GH, the maximal correlation was found at lag time 0 with a mean (95% confidence interval [CI]) correlation coefficient of 0.29 (0.22–0.37). All cross-correlations between lag time -90 and 80 were positive. The strongest correlation between cortisol and TSH was found at lag time 170 with a mean (95% CI) correlation coefficient of -0.30 (-0.36 to -0.25). Also, between lag times 90 and 180, cortisol and TSH were significantly negatively correlated, indicating that cortisol concentrations are negatively followed by TSH with a delay of 90 to 180 minutes. For ACTH and cortisol, the mean (95% CI) maximal correlation coefficient was 0.78 (0.74–0.81) at lag time 10, indicating that cortisol concentrations follow ACTH concentrations with a delay of 10 minutes. No significant cross-correlations between cortisol and GH, nor between ACTH and GH, were found. For ACTH and TSH, a weak maximal cross-correlation was observed at lag time 180 with a mean (95% CI) correlation coefficient of -0.19 (-0.24 to -0.15), which indicated that ACTH concentrations are negatively followed by TSH concentrations after 180 minutes.

### Cross-correlations stratified for lights-on and lights-off periods

**Figure 3.**
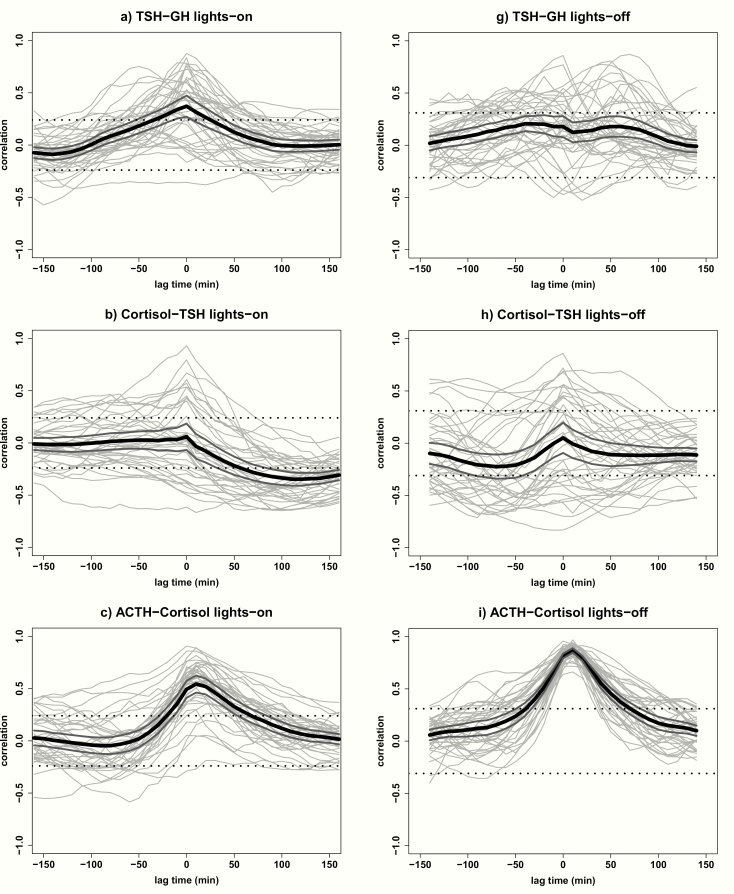

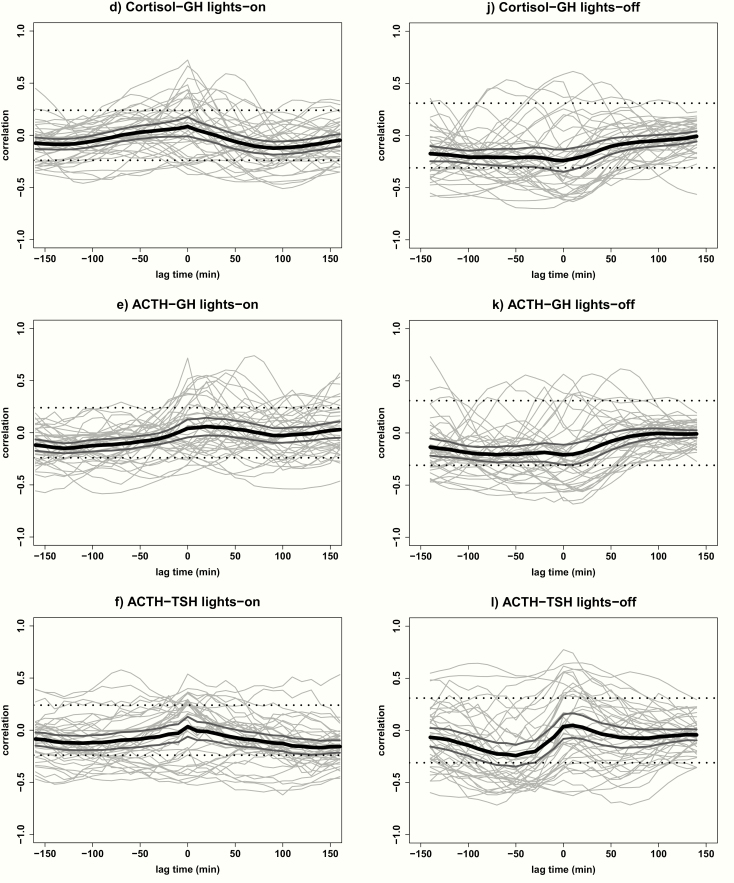
Cross-correlations of GH, TSH, ACTH, and cortisol stratified for lights-on and lights-off periods. Cross-correlations of hormone combinations of GH, TSH, ACTH, and cortisol in all 38 participants stratified for lights-on period (A-F) from 09:00 to 22:50 hours and lights-off period (G-L) from 23:00 to 08:00 hours. Cross-correlation assesses the relative strength between 2 hormone time series for all possible time shifts. The graph displays the correlation (y-axis) at a lag time in minutes (x-axis) with each gray line corresponding with 1 participant. The black line indicates the mean correlation for all participants and the 2 dark gray lines indicate the 95% confidence interval. The significance level is indicated by the 2 straight dotted lines at correlations -0.24 and +0.24 for the lights-on period and at -0.31 and +0.31 for the lights-off period. Negative lag times represent a correlation in which hormone 2 is followed by hormone 1; positive lag times represent a correlation in which hormone 1 is followed by hormone 2.

Figure 3 presents the cross-correlations of GH, TSH, ACTH, and cortisol stratified for lights-on and lights-off periods. In line with the correlation found between TSH and GH concentrations over the complete 24-hour period, we observed a strong positive correlation at lag time 0 (0.37 [0.27–0.47]) during the lights-on period. However, the correlation between TSH and GH disappeared in the lights-off period. Also, for cortisol and TSH, we observed similar results during the lights-on period as during the complete 24-hour period. A negative correlation at positives lag times was found during the lights-on period, but no significant correlation was found during the lights-off period. The cross-correlation between ACTH and cortisol is stronger during the lights-off period (0.87 [0.85–0.89]), than during the lights-on period (0.55 [0.46–0.63]). No significant cross-correlations between cortisol and GH, between ACTH and GH, and between ACTH and TSH concentrations, were found after stratifying the 24-hour data for lights-on and lights-off periods.

### Cross-correlations stratified for men and women

**Figure 4.**
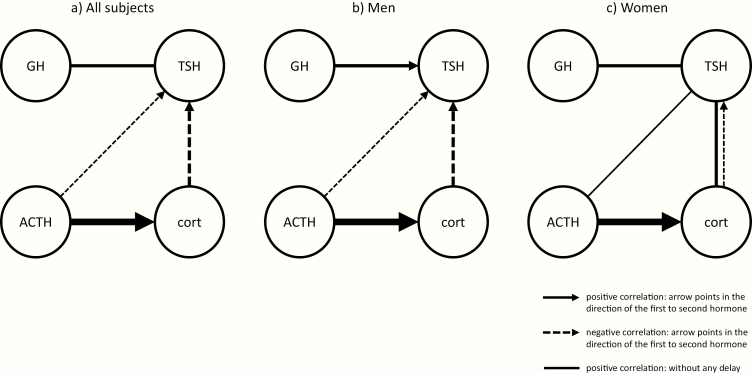
Summary of cross-correlations in (A) all subjects, (B) men, and (C) women. A graphical summary of cross-correlation analyses in (A) all 38 participants, (B) 20 male participants, and (C) 18 female participants. Solid lines represent positive correlations between hormones strongest at lag time 0, so without a delay. Solid arrows represent positive correlations between hormones which is strongest at a certain lag time, with the arrow directed toward the hormone that is following the leading hormone. Dotted arrows represent negative correlations between hormones strongest at a certain lag time, with the arrow directed toward the hormone which is (negatively) following the leading hormone. The weight of the line/arrow represents the strength of the correlation.

Cross-correlation results of GH, TSH, ACTH, and cortisol were stratified for men and women. In Figure 4, a graphical summary of main findings from cross-correlation analysis are displayed for all participants (A) and for men (B) and women (C) separately. For TSH and GH, the maximal correlation in women was found at lag time 0 with a mean (95% CI) correlation coefficient of 0.27 (0.15–0.39). In men, the strongest cross-correlation (0.33 [0.24–0.43]) between GH and TSH was found at lag time -40 indicating that TSH concentrations are following GH concentrations with a delay of 40 minutes. However, also in men there were positive correlations at all lag times between -100 and 120 minutes. The strongest correlation between cortisol and TSH in men (-0.35 [-0.42 to -0.28]) was found at lag time 170, but in women, the strongest cross-correlation (0.32 [0.20–0.44]) was found at lag time 0, indicating that cortisol and TSH concentrations were strongly positively correlated without a delay. However, also in women we observed a weak but significant negative correlation at lag times 120 until 180 minutes. For ACTH and cortisol, similar results were observed in men (0.78 [0.73–0.83]) and women (0.78 [0.73–0.82]). No significant cross-correlations between cortisol and GH, and between ACTH and GH, were found after stratifying for men and women. In men, a weak mean correlation coefficient of -0.21 (-0.27 to -0.15) at lag time 180 minutes was found between ACTH and TSH concentrations. In contrast, a weak positive correlation coefficient of 0.22 (0.11–0.33) was found at lag time 0 in women.

### Cross-correlations stratified for offspring and partners

When cross-correlation results were stratified for offspring and partners, similar results were observed in offspring (0.32 [0.21–0.44]) and partners (0.26 [0.16–0.35]) for the cross-correlation of TSH and GH concentrations (data not shown). Also, for cortisol and TSH, similar results were observed in offspring (-0.30 [-0.36 to -0.23]) and partners (-0.31 [-0.40 to -0.22]). The strongest cross-correlation coefficient for ACTH and cortisol concentrations in offspring was 0.75 (0.70–0.81), which was similar to their partners (0.80 [0.76–0.85]). No significant cross-correlations between cortisol and GH, and between ACTH and GH, were found after stratifying for offspring and partners. In partners, a mean negative correlation coefficient of -0.22 (-0.29 to -0.14) was found between ACTH and TSH concentrations. In contrast, no significant correlation between ACTH and TSH was observed in the offspring.

### Cross-ApEn of GH, TSH, ACTH, and cortisol

**Figure 5.**
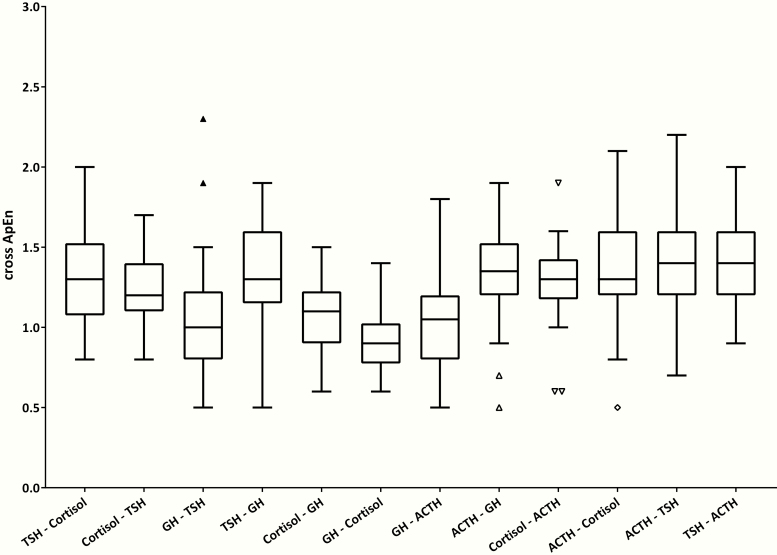
Cross-ApEn for GH, TSH, ACTH, and cortisol. Tukey box plots of the cross-ApEn results of combinations of the hormones GH, TSH, ACTH, and cortisol in all 38 participants. Lower cross-ApEn values signify greater synchrony between 2 hormone time series. Cross-ApEn, bivariate cross-approximate entropy.

Figure 5 presents box plots of cross-ApEn results for hormone combinations of GH, TSH, ACTH, and cortisol. Values of cross-ApEn ranged from 0.5 to 2.3 and mean values ranged from 0.9 to 1.4 in all participants, with lower cross-ApEn values signifying greater joint pattern synchrony between 2 hormone concentration time series. The cross-ApEn between GH and cortisol was the lowest of all hormone combinations with a mean (95% CI) of 0.9 (0.8–1.0). Also, the cross-ApEn values of GH-TSH, GH-ACTH, and cortisol-GH were lower than those of other hormone combinations. After stratifying for lights-on and lights-off periods, cross-ApEn values were significantly lower during the lights-on period compared with the lights-off period for the following hormone combinations: cortisol-TSH, GH-TSH, and GH-cortisol (data not shown). For cortisol-TSH, a mean (SE) difference of -0.17 (0.07) with a *P* value of 0.03 was found between lights-on and lights-off periods. The mean (SE) difference in cross-ApEn of GH-TSH was -0.21 (0.08) with *P =* 0.01 and for GH-cortisol, the mean (SE) difference was -0.15 (0.07) with *P =* 0.04. For the hormone combinations ACTH-GH, ACTH-cortisol, ACTH-TSH, and TSH-ACTH, cross-ApEn values were lower during the lights-off period compared with the lights-on period where lower cross-ApEn signifies stronger synchronicity. For ACTH-GH, the mean (SE) difference in cross-ApEn between lights-on and lights-off periods was 0.24 (0.06) with *P* < 0.001. The difference in cross-ApEn between lights-on and lights-off periods was greatest for ACTH-cortisol with a mean (SE) difference of 0.27 (0.07) and a significance of *P <* 0.001. A mean (SE) difference of 0.15 (0.07) (*P =* 0.03) for ACTH-TSH cross-ApEn values between lights-on and lights-off periods was found. Also, the cross-ApEn of TSH-ACTH was lower during the lights-off period compared with the lights-on period with a mean (SE) difference of 0.17 (0.07) and *P =* 0.02. No significant differences between men and women were found, but in general men tended to have lower cross-ApEn values than women (data not shown). Also, between offspring and partners no significant differences were observed (data not shown).

## Discussion

In this study, we aimed to determine the interrelationships between serum concentrations of GH, TSH, ACTH, and cortisol in healthy older individuals using 24-hour hormone concentration series with intervals of 10 minutes. We confirmed that ACTH is positively correlated with cortisol, where cortisol follows ACTH with a delay of 10 minutes (27–29). Previously, it was put forward that the synchrony between the ultradian rhythmicity of ACTH and cortisol is driven by an interplay between feedforward drive (of pulsatile ACTH on pulsatile cortisol secretion) and feedback inhibition (of pulsatile cortisol secretion on pulsatile ACTH secretion) (30). Here, we demonstrated that the cross-correlation between ACTH and cortisol was stronger during night hours. Furthermore, we corroborate previous observations that cortisol and TSH concentrations are negatively cross-correlated in healthy older individuals (8,31), where TSH follows cortisol concentrations with a delay of 170 minutes. Not earlier reported, a positive correlation between TSH and GH without any delay was found, which was more strong during daytime. The cross-ApEn analyses showed that GH and cortisol exhibit synchronous serum concentration patterns. Several differences in cross-ApEn values were found between lights-on and lights-off periods, indicating that the pattern synchronicity between hormones is dependent on the time of the day. No major differences in cross-correlations were found between men and women, except for the positive correlation without any delay between cortisol and TSH concentrations, which was found in women but not in men. In general, men tended to have lower cross-ApEn values than women, which was similar to other studies and could indicate that postmenopausal women have reduced hormone pattern synchrony compared with men (9,29). No major differences in the interrelationships between hormones were found between offspring of long-lived families and partners.

Although cross-correlation and cross-ApEn analyses are complementary methods, the strong cross-correlation found between concurrent GH and TSH concentrations were strengthened by a relatively low cross-ApEn of GH-TSH pointing to strong synchronization between the 2 hormone concentration series. This strong interrelationship can probably not be explained by circadian synchronicity because GH is mostly influenced by sleep and is less under circadian control (32). Both TSH and GH play important roles in the regulation of energy metabolism, growth, and development, which could explain the presence of the interrelationship between TSH and GH concentrations. Additionally, we could speculate that this interrelationship between TSH and GH is established by the dopaminergic or somatostatinergic system because these systems play regulatory roles in both the TSH and GH secretion (33). Moreover, TRH might stimulate, besides TSH, the secretion of GH, which was observed in a culture of rat cells (34). During the embryonic development of the anterior pituitary, specific genes direct the cells toward a particular fate. For lactotrophs, somatotrophs, and thyrotrophs, the same genes are involved in their development until the final differentiation. This means that lactotrophs, somatotrophs, and thyrotrophs largely share the same developmental cascade. Therefore, one might expect stronger correlations between TSH, GH, and prolactin than, for example, with ACTH or LH. This might explain the strong correlation between GH and TSH.

The strong negative cross-correlation between cortisol and TSH indicates that cortisol concentrations are negatively followed by TSH with a delay of 90 to 180 minutes. Literature shows that glucocorticoids indeed suppress TSH secretion, not only in patients with adrenal insufficiency but also in healthy young subjects (8,31,35,36). These studies were performed in young subjects, whereas we confirmed this observation in healthy older individuals. The cross-correlation as well as the pattern synchronicity were stronger during daytime than during nighttime. In line with this finding, administration of a cortisol-inhibitor (metyrapone) resulted in increased daytime TSH levels, whereas nighttime TSH levels did not alter, which might suggest that the peak in cortisol levels during early morning causes the daytime decrease in TSH levels (37). This inhibitory influence of cortisol on serum TSH levels might explain why the circadian rhythms of TSH and cortisol are out of phase; TSH has a nocturnal surge while cortisol has its nadir around that time and its peak in the early morning. Also, the interrelationship between GH-TSH was stronger during daytime than during nighttime. An explanation why some of the significant cross-correlations disappeared after stratifying for lights-off periods could be that there are less data points during the lights-off period. This dilutes any effects and increases the threshold value for significance. Interestingly, we observed an even stronger correlation between ACTH and cortisol during the lights-off period, which potentially could be explained by the fact that the ACTH-cortisol system is maximally active during nighttime. Similarly, cross-ApEn values of ACTH and cortisol were lower, indicating higher synchrony, during the night in this study, but also in healthy young subjects (27).

There is no cutoff value for significance for cross-ApEn values, but when comparing hormone pairs with each other, we found that the combination of GH and cortisol had the greatest joint pattern synchrony of all hormone combinations with higher synchrony during daytime. Another study found similar results for GH-cortisol cross-ApEn in healthy older men and women (9,38). Also, other studies have shown a link between cortisol and GH in human (39,40). In children, it has been shown that treatment with corticosteroids can lead to growth (hormone) inhibition (41). It is unknown whether such relationships change with aging. Aging could be seen as a state of chronic exposure to different forms of stress (including genotoxic stress and metabolic stress), which may affect cortisol, GH, and their interplay. Other cross-ApEn values of hormone combinations with GH were relatively low as well, including the GH-TSH and GH-ACTH combinations, which could indicate that GH is interlinked with many different hormonal axes. We did not find a significant cross-correlation between GH and cortisol, which demonstrates that cross-correlation and cross-ApEn analyses are complementary methods. Cross-correlation assesses the strength between paired time series for all possible time shifts, resulting in linear lag-specific correlations, whereas cross-ApEn quantifies joint pattern synchrony between paired time series, which is lag-independent (42). These methods therefore reflect different aspects of interrelationships between hormones.

This is 1 of the first studies in which interrelationships between hormones from different hypothalamic-pituitary-target gland axes over 24 hours in healthy older subjects have been investigated by cross-correlation and cross-ApEn analyses. This innovative approach is a strength of the study although it makes it exploratory in nature because its novelty limits the availability of similar studies. Cross-ApEn and cross-correlations values changed within obese patients with polycystic ovary syndrome after a low caloric diet, which shows that these analyses are sensitive to detect alterations after for example an intervention (43). Cross-correlations between hormone concentrations are not evidence for a direct causal relationship between hormones. Furthermore, the potential day-to-day intrasubject variation remains unknown. Cross-ApEn is a validated tool to determine the joint pattern synchrony in a closed hormone system with known feedforward and feedback signals. However, cross-ApEn is rarely applied to combinations of hormones from different hormonal axes, which makes it harder to interpret the biological meaning. Therefore, this study is more descriptive than conclusive. Nonetheless, it may promote the generation of new hypotheses on which future research can build.

There are many modulating factors for hormone secretion, including sleep, BMI, sex, and age (44). Age-related changes in hormone secretion are mostly observable in the regularity of secretion and in the amplitude and circadian timing of the 24-hour rhythm (45,46). For example, for cortisol, mean levels were higher, but the relative amplitude was lower in older subjects than in young subjects (46–48). Higher cortisol levels were observed in the evening in older subjects compared with young subjects (46,47), but 1 study (49) showed that this was not the case in subjects up to 64 years of age. There are also indications that a phase shift in circadian timing occurs with aging (46–48). Furthermore, the feedback inhibition of cortisol on the secretion of ACTH was decreased in old subjects compared with young subjects (50). Also, the regularity of cortisol secretion, assessed by ApEn, was decreased with age, as well as the regularity of TSH and GH secretion (44,46,51). Furthermore, GH secretion decreases with age and the onset of the nocturnal surge of TSH was advanced by increasing age (51,52). Whether the interrelationships between hormones alters with aging is not known. Nevertheless, because regularity, circadian timing, and feedback mechanisms of hormones alter with age, one could hypothesize that the synchronicity and cross-correlation between hormones would decrease with aging. Although we did not include individuals with a large range of chronological age, we did include offspring of long-lived families and their partners. It is assumed that offspring of long-lived families are biologically younger than their partners because, among the key findings from the Leiden Longevity Study, were the observations that the offspring had lower prevalence of myocardial infarctions, diabetes mellitus, hypertension, and metabolic syndrome compared with their partners (12,53). Therefore, we hypothesized that the offspring of long-lived families would have stronger hormonal interrelationships than controls. However, no major differences in the interrelationships between hormones were found between offspring and partners. This could indicate that this interplay between hormones is crucial for survival and if this interconnection would disappear, it would lead to illness. Participants in this study were selected based on their health status, which resulted in a group of healthy older individuals and this could have influenced the results.

Hormones of the hypothalamic-pituitary-gonadal and the hypothalamic-pituitary-prolactin axes were not taken into account in this article. However, GH, TSH, ACTH, and cortisol might also interact with these hormones, including LH, testosterone, estrogen, and prolactin. However, FSH might not be suitable to include in these types of studies because of its long half-life. Lactotrophs, somatotrophs, and thyrotrophs largely share the same developmental cascade. Indeed, studies showed a positive association between the hypothalamic-pituitary-thyroid axis and prolactin; TRH regulates the synthesis and release of prolactin (54,55), and Saini *et al.* found concurrent pulses of TSH and prolactin in young men (56). Furthermore, prolactin was positively correlated with GH, TSH, and ACTH without any delay and with cortisol at a lag of 10 minutes in obese women (7). Also, the hypothalamic-pituitary-gonadal axis seems to be associated with other hormonal axes; long-term testosterone administration resulted in increased overnight GH secretion in healthy older men (57) and prolactin concentrations increased in response to estrogen treatment in postmenopausal women (58).

## Conclusions

This study demonstrates that interrelations between hormones from interlinked as well as different hypothalamic-pituitary-target gland axes are observed in (older) individuals. In particular, the correlations between cortisol and TSH concentrations, between TSH and GH concentrations, and the great joint pattern synchrony between GH and cortisol, are indications that distinct hormonal axes interact in healthy older individuals. No major differences were found between men and women, except for the positive correlations between cortisol and TSH concentrations found in women. Also, no major differences between offspring of long-lived families and partners were found. The cross-correlation and pattern synchronicity between TSH and GH, and the pattern synchronicity between cortisol and TSH, were stronger during daytime than during nighttime, but the cross-correlation and pattern synchronicity between ACTH and cortisol were stronger during nighttime. More research is needed to determine the biological meaning and clinical consequences of these interrelationships between pituitary hormones.

## Abbreviations

ApEn: cross-approximate entropy
BMI: body mass index
CI: confidence interval
cross-ApEn: bivariate cross-approximate entropy
RRID: Research Resource Identifier

## Additional Information

***Data Sharing Statement:*** The datasets generated during and/or analyzed during the current study are not publicly available but are available from the corresponding author on reasonable request.

***Disclosure Summary:*** The authors have nothing to disclose.

***Data Availability:*** The datasets generated during and/or analyzed during the current study are not publicly available but are available from the corresponding author on reasonable request.
